# Taking time: The temporal politics of dementia, care and support in the neighbourhood

**DOI:** 10.1111/1467-9566.13524

**Published:** 2022-09-05

**Authors:** Richard Ward, Kirstein Rummery, Elzana Odzakovic, Kainde Manji, Agneta Kullberg, John Keady, Andrew Clark, Sarah Campbell

**Affiliations:** ^1^ Faculty of Social Sciences University of Stirling Stirling Scotland; ^2^ School of Health Sciences Jonkoping University Jonkoping Sweden; ^3^ Independent Researcher (previously Faculty of Social Sciences University of Stirling) Stirling Scotland; ^4^ Faculty of Medicine and Health Sciences Linköping University Linkoping Sweden; ^5^ Division of Nursing Midwifery and Social Work University of Manchester Manchester UK; ^6^ School of Health and Society University of Salford Salford UK; ^7^ Department of Social Care and Social Work Manchester Metropolitan University Manchester UK

**Keywords:** austerity, care, dementia, social care, time

## Abstract

Dementia is a global health challenge and currently the focus of a coordinated international response articulated through the notion of ‘dementia‐friendly communities and initiatives’ (DFCIs). Yet, while increasing research attention has been paid to the social and spatial dimensions to life with dementia in a neighbourhood setting, the temporalities of dementia have been largely overlooked. This article sets out different aspects of the lived experience of time for people with dementia and unpaid carers, before exploring the temporal politics of formal dementia care and support. The authors show that time is a site for material struggle and a marker of unequal relations of power. People with dementia and unpaid carers are disempowered through access to formal care, and this is illustrated in their loss of (temporal) autonomy and limited options for changing the conditions of the care received. The authors advocate for a time‐space configured understanding of the relationship with neighbourhood and foreground a tempo‐material understanding of dementia. Set against the backdrop of austerity policy in the UK, the findings reveal that ongoing budgetary restrictions have diminished the capacity for social care to mediate in questions of social justice and inequality, at times even compounding inequity.

## INTRODUCTION

Dementia is now widely recognised as a global health challenge. An estimated 50 million people are currently living with dementia worldwide and this number is estimated to reach 82 million by 2030 and 152 million by 2050 (WHO, 2017). These projections alone have shaped the momentum of our response to the condition. Additionally, the evolving coronavirus pandemic has exposed entrenched inequities in the care system while underlining the importance of recognising global connectedness and interdependencies with respect to health. The pandemic thus lends further urgency to co‐ordination of an international response to dementia that (pre‐COVID) had already begun to coalesce around a place‐based agenda articulated through the notion of ‘dementia‐friendly communities and initiatives’ (DFCIs) (see ADI, 2017; WHO, 2021).

An emerging focus on dementia in the neighbourhood represents a shift in thinking and approach in three key respects. First, it marks an alternative geography and spatiality of dementia as care and support migrate from institutionalised settings (Blackman et al., 2003; Burton & Mitchell, 2006). Second, it foregrounds new and emerging socialities through a collectivised response to the condition. At least in theory, responsibility for support now rests with a broader range of neighbourhood‐based actors from local government, the cultural sector, sport and leisure, retail, education, banking, business, transport and tourism (e.g. Brorsson, 2021; Connell & Page, 2021; Edwards et al., 2018; Fortune & McKeown, 2016; Risser et al., 2015). Third, it heralds a shifting temporal order. An accompanying emphasis on ‘living well with dementia’ (Department of Health, 2005) foregrounds chronicity, challenging earlier nihilism within health care wherein a diagnosis was frequently considered the beginning of the end. Yet, while the spatial and social dimensions of this developing global response have attracted increasing attention in research (see reviews by Gan et al., 2021; Li et al., 2021 and Sturge et al., 2021), far less concern has been shown for the temporalities of dementia.

In this article, we consider the relationship of people living with dementia to time (i.e. temporality) and the temporal politics of care and support. Drawing on empirical research, we advocate a space‐time configured understanding of people’s relationship to their neighbourhood that underlines the particularities of place, as a critical response to the generalities of an emerging global DFCI movement. Our findings suggest that the language of positivity and progress in which dementia‐friendliness has been cast (policy aspirations for dementia‐future), mask the impact of austerity and a receding state upon the lives of people with dementia (the lived experience of dementia‐present). We show that a focus on the temporalities of dementia not only sheds light on the shortcomings of the care system but reveals that time itself is a significant site of material struggle, where control over the temporal ordering of care is a clear manifestation of unequal relations of power.

## BACKGROUND: DEMENTIA AND TIME

In the UK, the drive for ‘living well’ aimed at promoting integration and consensus‐building across dementia care services (Banerjee, 2010). It has subsequently served to anchor a discourse of dementia‐friendliness as a hoped‐for outcome to community development initiatives (Darlington et al., 2021). Notions of ‘living well’ (Martyr et al., 2018) and ‘positively’ (Wolverson et al., 2016) with dementia echo more established arguments for successful and positive ageing and for positive living with chronic illness and disability (e.g. Mudge et al., 2013), which have themselves been critiqued for imposing hegemonic temporalities on a global scale (e.g. Benton et al., 2017; Gibbons, 2016). The idea of living well expands opportunities for post‐diagnostic intervention, combining with pre‐diagnostic identification of biomarkers and introduction of the category of mild cognitive impairment to extend the temporal reach of ‘biopower’ (Foucault, 1998) as a greater portion of people’s lives become knowable and hence more readily governable (Tomkow, 2020). Nonetheless, while the call for living well may substitute therapeutic nihilism with newly realised chronicity, it remains open to the charge of othering those who fail at doing so (McParland et al., 2017). The idea thus carries moral connotations, placing responsibility on the individual and in common with positive ageing, hails the person with dementia as a responsible citizen, where living well is commensurate with making fewer demands upon the state (Sandberg & Marshall, 2017).

Under the banner of ‘living well’ and ‘dementia‐friendliness’, neighbourhoods have become a locus for interventions including a range of educational and training programmes aimed at supporting resilience, self‐care, self‐management and peer support (Keyes et al., 2016; Mountain & Craig, 2012; Quinn et al., 2016; Teahan et al., 2018). The push for dementia‐friendly communities places responsibility on people with dementia and unpaid carers to appropriate (assumed) resources and capital available to them in their proximate environment to make life with dementia more manageable (Ward et al., 2021b). From this perspective, the dementia‐friendly agenda, like the age‐friendly movement on which it has been modelled, might be understood as a vehicle for shifting a duty of care from institutions of the state onto local communities, informal support networks and ultimately, the individual (Joy, 2018). Yet, efforts at coordination and standardisation of provision (e.g. WHO, 2021) appear to transcend time and space, glossing over the contested nature of place while failing to recognise differences attached to relations of gender, class, race, age, sexuality etc. In its increasingly global formulation, dementia‐friendliness thereby neglects the contingent and fluid nature of everyday living and precarity in the situation of many of those affected by dementia (Grenier et al., 2017), so vividly underlined by the unfolding pandemic.

In a recent review, Eriksen et al. (2020) found an overall inattention to the experience of time in research on dementia. Their findings revealed how people living with dementia engaged with dimensions of time, including future uncertainty. A small body of research has considered the significance of time for dementia care. Thus, Nygard and Johansson (2001) have explored efforts by people with dementia to overcome certain difficulties, such as taking more time to accomplish tasks and employing ‘time aids’ such as calendars and diaries. The authors point to the relational nature of time in the context of caring relationships as couples accommodate to one another’s rhythms. In a residential care context, Kitwood’s (1997) notion of outpacing directs attention to temporal struggle as a symptom of malignant social psychology, wherein workers undertake care at a rate the person with dementia is unable to keep up with. Outpacing shows how unequal relations are upheld temporally, becoming a source of social exclusion. By contrast, Egede‐Nissen et al. (2013) note efforts by care workers to resist the ‘metric straight‐jacket’ imposed within residential care through practices aimed at keeping pace with the person with dementia. These practices are part of a temporal culture that the authors suggest reveal the ‘time ethics’ of dementia care. Gjodsbol and Svendsen (2019) similarly focus on temporal practices at both early and late points in dementia where practitioners engage in ‘timework’ to uphold personhood. By drawing upon the biographies of individuals in the course of care provision, workers reject the biomedical ‘prophecy of decline’ (p. 54, 2019).

Dementia care has been argued to encompass past, present and future‐oriented approaches (Eriksen et al., 2020). Interventions such as reminiscence, reality‐orientation and living wills are temporally oriented and yet rarely explicitly engage with or problematise underlying notions of time. Narrative interventions, which focus on sense‐making through temporal (re‐)ordering, can support a person to locate a sense of self in and through time while informing care practice (e.g. Scherrer et al., 2014). While Eriksen et al. (2020) suggest this (re‐)representation of self constitutes an example of ‘lived time’, other commentators have pointed to the way that narrative approaches can strip away the more visceral and chaotic immediacy of temporal experience. For example, Changfoot et al. (2021) point to the cyclical and rhizomatic experience of time as past, present and future fold into one another as we age or live with conditions such as dementia. Kafer (2021) is similarly critical of the largely unexamined way that illness narratives ‘rely on the straightness of linear time’ (p. 417) and the cultural conventions that shape their production. As such, it is important to acknowledge how cultural intelligibility shapes the narrativising of dementia and not lose sight of the embodied, affective and material experience of time.

Like many chronic and progressive conditions, dementia can alter a person’s relationship with time but there is also evidence that the nature of impairment associated with conditions such as Alzheimer’s directly affects the perception of time. As such, orientation to time has long served as a guide to the presence and severity of cognitive impairment (O’Keeffe et al., 2011). Damage to the hippocampus (part of the cerebral cortex) can create difficulties in judging duration (the estimation of time passed) and compromise a person’s capacity to recall and relive past events. This, combined with difficulties in creating new memories, has led to descriptions of people with dementia as ‘stuck in time’ (El Haj and Kapogiannis, 2016). Such biomedical framing rests upon an unexamined construct of time as singular, linear and objectively quantifiable and has led to some of the starkest representations of the condition. Graphs used to depict the progression of dementia, where the *X*‐axis denotes time and the *Y*‐axis functionality, use a single downward line (a stepped line in the case of cardiovascular dementia) that equates time passing to unrelenting decline (e.g. Treiber et al., 2011). We know from accounts offered by people with dementia how such visualisations translate into health‐care practice. For example, the activist Kate Swaffer described receipt of a diagnosis in her late 40’s as an instance of ‘prescribed disengagement’, where people with dementia are advised to plan for their demise: ‘this sets us up to live a life without hope or any sense of a future’ (p. 3, 2015).

This struggle over the potential to imagine a future with dementia resonates with broader encounters with ‘foreclosed futures’ (Kafer, 2013) in the biomedicalisation of illness and disability. Yet, while research has helped to understand adjustment to a diagnosis over time (Vernooij‐Dassen et al., 2006), few studies have sought to critique this temporal framing, despite widespread recognition of the impact on timing and disclosure of a diagnosis and availability of post‐diagnostic support (Cations et al., 2018). As such, Ward (2016) argues that much can be learnt from notions of crip time and queer time in opening‐up dementia futures as a field of resistance to normative temporalities. For example, Halberstam (2005), who focussed on the abbreviated life course of people with HIV/AIDS prior to retroviral therapies, noted how queer subcultures fostered new and alternative temporalities, outside of heteronormative constructs of ‘reproductive and family time’. Such exclusionary temporalities are also problematised in Sandberg and Marshall’s (2017) critique of policy discourses of ‘successful’ and ‘positive ageing’ which rest upon the implicit othering of those considered to have aged unsuccessfully, including individuals with dementia. In this context, orientation to the idea of ‘living well with dementia’ in health policy (DoH, 2009) is notable for mimicking a similar trope in policy discourses on ageing, while contesting their implied othering of dementia.

## THE RESEARCH

The ‘Neighbourhoods: our people, our places’ (NOPOP) study (2014–2019) was undertaken as part of a wider programme of research exploring dementia and neighbourhoods (Keady, 2014). Our aim was to investigate how neighbourhoods can support people living with dementia to remain socially and physically active. The project extended over three fieldsites: Greater Manchester in northern England; the Central Belt of Scotland; and the county of Östergötland in the south of Sweden. Across all three fieldsites, walking interviews were used to engage in ‘in‐situ’ place‐oriented discussions (see Odzakovic et al., 2020 for further discussion). The UK fieldsites also employed home tours, drawing upon Pink’s (2009) ‘walking with video’ method, where participants took us on a tour of their domestic spaces. Additionally, we used social network mapping to explore connectedness and the everyday give and take of help and support (see Campbell et al., 2019 for more detail). Returning to many of our participants after a break of 8–12 months, we repeated the walking and mapping interviews (please see Table 1 for participant profile and methods). For this article, our analysis focuses mainly on the walking interviews and network mappings (the latter were conducted in the UK fieldsites only).

**TABLE 1:** Participant profile and methods

		England	Scotland	Sweden	Total
**Participants**		54	47	26	**127 participants**
	Living with dementia	29	22	16	67
	Nominated care‐partner	25	25	10	60
	Living in couple dyad	50	32	20	102
	Living alone	4 (PwD)	6 (PwD)	6	25
			9 (carer)		
**Age** (of a person living with dementia)	Youngest	57	51	62	
	Oldest	88	88	87	
**Methods**	Network maps	53	55	30 (sit‐down interview)	138
	Walking interviews	41	40	18	99
	Home tour	30	29 (not all filmed)	0	59
	Other	2 mobility diaries	5 mobility diaries, 1 diary	0	8
	Total	126	130	48	**Total 304 interviews**

*Ethics*: Ethical approval was obtained from the NHS Health and Social Care Research Ethics Committee in the UK and the Regional Ethical Review Board in Linköping (the county of Östergötland, Sweden). We followed a process consent approach (Dewing, 2008) and were guided throughout by the provisions of the Mental Capacity Act (2005) and the Adults with Incapacity Act (Scotland) (2000).

### Insights from the temporalities of research

Two points for data capture allowed us to track people’s evolving experience of living with a progressive condition. We learnt that while some people’s lives underwent radical change, others had managed to maintain continuity, often through ‘holding onto familiarity’ (Ward et al., 2021b). Such variation revealed there was no common temporal frame to life with dementia, that a biomedical ‘prophecy of decline’ was unrepresentative of the diversity of experience and that this diversity signalled a multiplicity of temporalities.

Walking with participants animated interviews offering insights into the space‐time configurations of people’s lives that would have been inaccessible using sedentary methods, we learnt how people used time and where time was spent. Network mapping revealed the temporally mediated nature of relationships. For example, we learnt how bonds grew over time, the value placed on investing time in relationships, of giving time to people, sharing and saving time. The network maps told their own story as much for who was left out as for who was included. Interviewing thus provided a clear sense of the ‘*chosen neighbourhood*’ (akin to Donovan et al.’s (2003) ‘chosen families’) produced in time and space; those people and places that served as coordinates of everyday life.

## FINDINGS

In this section, we present and discuss our findings, first considering the everyday temporal experience of dementia and care before considering how access to formal care and support fits into this broader picture.

### The lived experience of time with dementia

Many participants described dementia as temporally framed. Rather than a continual presence in their lives, it felt more like points of intrusion that shifted and disrupted day‐to‐day experience. Some referred to moments of confusion or disorientation as a ‘glitch’ or ‘blip’ or like a fog descending, often in response to particular conditions. As such, dementia was configured in time and space and frequently appeared as a contingent effect of specific material relations (Schillmeier, 2008). For instance, one of the few occasions when a participant became lost during our walking interviews was upon encountering pavement repairs that required a detour. It was an unexpected change that disrupted an engrained relationship with the local environment. We heard of many similar moments when a familiar situation or setting was suddenly rendered unfamiliar. In his study of the neighbourhood experiences of people with visual impairment, Schillmeier shares a case study of a woman who is blind standing at the door of a supermarket that has recently altered its layout. The memorised space of the shop floor no longer synchronises with the sensed space before her; ‘she experiences the *no longer* and *not yet*’ (p. 223). Schillmeier uses this example to build a case for a temporal conception of dis/ability arguing that independence is a ‘fragile tempo‐material achievement’ (p. 217) …‘easily disturbed when bodies, technologies and things do not assemble properly’ (2008, p. 218). This temporalised understanding of dis/ability resonates with the experiences of the people with dementia we interviewed. The unexpected changes that led to a person’s relationship to place breaking down revealed the significance of habituated spatial practices that had evolved over time, as much as the more immediate multi‐sensory engagement with the present neighbourhood. Such a temporalised conception of dementia points to an experience of dis/ability or in/capacity that is fluid and shifting rather than as stable and which persists in continuous time.

In practical terms, this shifting experience led to an intermittent requirement of having to ask for help. Nonetheless, participants spoke of self‐limiting their call upon the time of others, revealing an awareness of their interconnectedness with a wider network and of how the use and management of time can diminish the time of others. Ruth described the tensions underlying help‐seeking:

I’m not good at asking… to ask for help. And it can be a brush off you know, in a very busy [way], it makes it harder to ask again. And you’re constantly waiting on people finding time for you … “I wonder if you could find time?” – “Oh yeah, yeah, no bother” and then you know it never happens and you’re like… I wait, two weeks go by and then you gently ask and then it happens

Encounters like this hint at the way dementia policy seeps into everyday situations as a discourse of dementia‐friendliness promotes growing reliance on discretionary help in substitution for entitlement to state support (Shakespeare et al., 2019). While such exchanges may seem unremarkable and even mundane, they ultimately shape the sociality of the neighbourhood and how the person with dementia is positioned within this. In her analysis of the cultural politics of time, Sharma (2014) draws attention to what she describes as ‘the different temporal itineraries that constitute social space’ (p. 5). Sharma suggests that encounters with others (be it individuals, agencies, institutions or the state) inevitably involve differentials in power, and these are both marked and negotiated through a process of ‘recalibration’ where one party speeds up or slows down to synchronise with the other. For instance, we can see in Ruth’s account above how she is continually required to adjust to various helpers’ temporalities. In this way a focus on time assists in understanding power as an active, rather than a static challenge in everyday relations, and one that is often worked out temporally.

The requirement to recalibrate and synchronise with others could at times prove challenging when a person needed to perform a particular task in a public setting. Under scrutiny from others, material and social conditions often combined to create a sense of taking too much time. Discomfort paying at the shop till was frequently cited, while Betty (care partner) recalled a hurtful incident at the local social club where her husband had struggled to keep up with the bingo caller drawing whispered comments from other members. Such experiences could lead to avoidance of certain venues or situations, but we also heard how people sought out temporally inclusive settings including green spaces and certain retail and recreational destinations. This performative aspect to time as pace or tempo could lead to becoming visible as a person with dementia (Fletcher, 2019) and attract a stigmatising response, albeit sometimes just a collective stare (van Wijngaarden et al., 2019). Talking of the related experience of crip time Kafer (2021) makes the point that the temporalities of disabled people are often homogenised, perceived as a generalised slowness that marks a refusal to tolerate or even acknowledge a multiplicity of temporalities in public spaces.

Our research highlights the interlocking and intertwined temporalities that frame caring relationships. Yet, being part of such a relationship often led to starkly differing temporal experiences and outcomes for people with dementia compared to their care partners. A common experience for people with dementia was a struggle to fill time. For instance, Sean passed his day by touring the charity shops that punctuated his local high street. As useful examples of more temporally inclusive retail spaces, the shops allowed him to browse, to handle merchandise and to chat with staff. In this way Sean staved off the potential for otherwise prolonged periods of under‐occupation. By contrast care partners reported a struggle to make time. Indeed, care itself was often temporally defined, involving questions of how time is used, tensions between competing temporal demands and an embodied urgency and press of time. Investing time to care inevitably led to absences from other relationships. Earlier studies of informal dementia care have highlighted time devoted simply to having a presence for purposes of ‘supervision and surveillance’ (Wimo et al., 2002). Baraitser (2017) describes these ways of using time as durational practices that keep things going and makes the point that what may appear as leisure time for carers is often ‘part of maintaining the supportive structures in which [care] can remain viable’ (p.73).

Making a case for temporal justice, Goodin (2010) points to the unequal social distribution of ‘discretionary time’, that is, time over which people have a degree of autonomy after working commitments, self‐care and care responsibilities for others are dispensed. Goodin argues that social policy has a redistributive role to play. However, such calls for policy intervention have been critiqued for treating discretionary time as individualised and readily quantifiable. Tyssedal (2021) points instead to the situatedness of time, arguing that efforts to compare and measure free time, as a route to addressing injustice, fail to consider its use value. For instance, discretionary time may coincide with low energy or exhaustion (the case for many of the care partners we spoke to), it may not be aligned with the freetime of others, or there may be limited prospects for how time can be used, as revealed by many of the people with dementia we interviewed. We have discussed elsewhere (Odzakovic et al., 2021) descriptions of the ‘quiet neighbourhood’ where people with dementia often felt isolated as friends and neighbours departed for work or education. Synchrony of shared time was thus integral to opportunities for sociability (Clark et al., 2020). We also saw the value to a sense of belonging from entrainment to the broader collective rhythms of the neighbourhood (Ward et al., 2021a).

Both Baraitser (2017) and Sharma (2014) have argued for recognising the relative worth attached to different experiences of time, and how sped‐up, productive temporalities attract greater interest and esteem than time that drags, or which leads to no tangible output as for those living with chronic illness or who care for others. Such experience of time often remains hidden, suggesting political disinvestment (Sharma, 2014). Baraitser has a particular concern with those for whom ‘time fails to unfold’ (i.e., ‘unbecoming time’), showing that an experience of time is not only an outcome of a person’s social situation but integral to the ongoing and negotiated production of disadvantage and privilege. At the heart of her argument lies an emphasis on ‘duration not difference’ whereby a focus on the lived experience of time can provide a unique way of understanding how relations of power situate different groups and individuals according to a broader social ordering of temporal worth.

### The dominance of service time

A policy of austerity was formally introduced in the UK by a coalition government following the 2008/9 recession, proposing £83 billion of cuts by 2015, including £8 billion in cuts to the social care budget, with funding for social care for people aged over 65 particularly hard hit, falling by 21% between 2009/10 and 2015/16 (Hastings et al., 2015). The NOPOP study commenced in 2014 with much of our interviewing taking place between 2016 and 2018. In addition to witnessing the impact of incremental cuts to services and state retrenchment, we learnt that time itself is a battleground for autonomy linked to hierarchies of temporal worth.

Access to formal care often led to surrendering temporal autonomy. Decisions over the frequency and duration of support were largely out of the hands of service recipients. A common theme from our discussions was the struggle over what constituted ‘enough time’ revealing that care is frequently under‐timed in a context where time itself has been monetised within the care system. Despite a wealth of evidence from feminist analyses of care and body work that demonstrate the inadequacy of a chronometric regime (e.g. Cohen, 2011; Davies, 1994), providers still allocate care on the basis of standard ‘clock‐time’ measures of tasks. Indeed, some participants referred to the input of homecare in exclusively temporal terms: ‘We get a 45, two 15’s and a 30’ (Siobhan carer to her mother Kathleen). The consequences of under‐timing were social, material, spatial and corporeal. For instance, Siobhan noted the gustatory and nutritional inadequacy of mealtime visits: *‘On a weekend she wants sausage, black pudding, bacon and eggs. That’s what she wants but they won’t do that. So, she gets a piece of toast. Lunchtime she gets a microwave meal. Evening she gets a microwave meal… and she’ll say ‘I’m fed up with this damn microwave food’.*

Tightly timed visits limited opportunities for sociability but also produced particular experiences of time as care recipients were required to recalibrate to the temporalities of workers. Edith, who lived alone said of one worker: ‘*One came in that said, “right, take those tablets and I’ll put out the afternoon tablets and you can just take them yourself, because I’m really busy”… and I said* “*well the main reason for you coming in is to make sure my tablets are right*”. *And she said “och, you’ll do okay*”’. No time was allocated for workers to support people with dementia to leave their home and journey into the neighbourhood, revealing a temporal dimension to isolation and reinforcing the potential for under‐occupation as we outlined above. The hidden time costs to carers included repeatedly inducting new workers in a context of churn within the sector, filling‐in when workers arrived late or not at all and restoring order after a visit. Delia faced an additional cultural time penalty, making time to teach workers how to prepare suitable meals for her mother: ‘*Mum has always enjoyed different foods… but they see an elderly Caribbean woman and they think* “*stereotype*”’. The cumulative impact of these largely hidden but manifold time penalties was to intensify rather than alleviate the time pressure upon carers.

Delia was one of a number of carers who fought to resist the temporalities of formal care. Realising complaint was fruitless, she negotiated with a home care worker to provide additional hours of support to her mother in a private arrangement between the two women. We learnt how a number of care dyads similarly fostered affinities with particular workers in this way. While often cast as friendships these relations had a clearly strategic purpose in keeping care viable and signal largely unseen processes of temporal resistance, achieved through informal negotiation at the edges of the care system. The implications of under‐timing for workers have been outlined by Baines et al. (2021), who note they are often manoeuvred into ‘compulsory time philanthropy’ to meet their commitment to clients. Hayes and Moore (2017) hint at the temporal infrastructure that creates these conditions, including practices of ‘cramming’ whereby a worker’s rota is knowingly filled with more visits than can be properly serviced.

A receding state had altered the role and perception of public sector practitioners. Jayne, who cared for her husband up to and after his admission to residential care, was one of the very few participants to put a public sector practitioner on her social network map. She noted warmly that her community psychiatric nurse (CPN) had continued to visit after her husband’s admission, recognising her ongoing support needs, but had been put under increasing pressure to close the case:

She was told to, that she didn’t need to come any more and I just felt as if like you’d had your throat cut. It was like, oh it was awful. And she said “no”, she said “I’m still going to come and see you”.

This was just one instance where we heard of individual practitioners pushing back at the mounting temporal restraints imposed upon them. It also reveals how service provision can erode a person’s sense of (temporal) worth. In a context of tension between time‐consuming paperwork and a ‘compassionate temporality’ focussed on service users (Yuill & Mueller‐Hirth, 2019), we learnt that public sector practitioners are becoming increasingly hard‐to‐reach, their support ever‐more reactive as contact time reduces. For instance, Malcolm reflected that no one had noticed his wife’s decline into depression until they had reached crisis point. Our findings reveal that institutional timeframes consistently overshadow ‘temporalised need’ (i.e. need as it occurs in time) linked to the shifting nature of dementia that we outlined earlier.

Pressure to reduce budgets and find efficiencies in provision had led to closure of small‐scale localised services in favour of creating more centralised support hubs. These local losses often prompted informal cost‐benefit analyses as travel time was weighed against the rewards of attending support groups, or social activities. For David, the time spent driving his partner Florence to a day centre negated any potential respite benefit and led to his decision to decline the service. We heard of many further instances where time penalties and cost led to service refusal or withdrawal, highlighting how ‘time‐taking’ leads to multiple material and social losses. Phillipa (care partner to Thomas) revealed the layered impact of service reductions when accessing a dial‐a‐ride service to reach a support group:

Well the timing, we’ve tried [but] they can’t pick you up at the time that you want it’s either earlier or later and you get there and they may not be able to pick you up or bring you back so you just don’t ring really… it was after the time that they work so it was 4 o’clock finish and they said that was too late they’ve got to be off the road. It was across boundaries.

The couple’s predicament reveals the complexities in challenging conditions where services are outsourced to multiple providers, all of which individually enact reductions that pass on material and space‐time costs that ultimately compound isolation and under‐occupation.

Incremental reductions and temporal rationing of support were markers of the cuts to social care budgets taking effect. Often rationing was premised on shifting definitions or categories of eligibility as Sean’s experience demonstrated. Having described his initial experience of formal support: ‘*They came and took me out places*’ he recalled a gradual whittling, until eventually no longer being eligible for assistance:

I’m regarded [now] as self‐supporting. So, somebody’s sitting at a desk, they’ve never met me, they’ve never seen the house… They call me self‐supporting. No, self‐managing. I think it’s self‐management. But I don’t manage […] [My wife] manages my medicine. She puts it in boxes for me and arranges financial things.

In a later interview with both Sean and his wife, Frances, we were able to hear her experience of this service withdrawal:

I just feel sometimes I’m getting kind of bogged down again, tied up with things. I’m not getting… doing things for myself that I’d hoped to do […] And my daughter’s in London with a baby and I’m getting that I don’t even have like free weeks that I can say “well I’m going down to London” because this is on, that’s on, he’s got appointments at doctor’s clinics, plus all the other things.

Frances’ dilemma indicates how support networks evolve over time, where for instance, the arrival of a newborn brings new bonds and commitments. Yet, as she is compelled to devote more time to supporting her husband, she has less for her daughter and grandchild, revealing how the outcome of Sean’s needs assessment had rippled outward into the wider network to which he belongs. A focus on time helps to foreground the relationality of care, but also the absence of accountability by providers to this wider web of support.

Waiting has garnered particular interest in the sociology of time, not only for the experience of time it induces (Bissell, 2007) but also as an indicator of inequity (Lee et al., 2020). In a context of austerity‐hit health and social care provision, commentators have argued that waiting and delays are intentionally punitive; a knowing ploy to remind those reliant upon support of their powerlessness and low temporal worth (e.g. Kiely, 2021). In our study, waiting was an almost universally shared aspect of accessing formal support that produced a visceral awareness of time passing. Participants described suspension in time while applications were processed, or decisions reached. Susan, with a diagnosis of young onset dementia, pointed to an unwanted embodied sense of ‘unbecoming time’ as she shared her mounting dread and trepidation while awaiting a benefits assessment, knowing this could lead to a reduction or loss of income (which it ultimately did). Jo, whose benefits were frozen following her partners admission to residential care reported: *I’m living out of my freezer, now … [I’m] even turning the electric off, you know, because of the heating, because I’m thinking, well, at the moment, I don’t know.* In low‐income households, a freezer full of food can serve as a final buffer from poverty and reliance on food banks. Waiting and delays thus had directly material and financial implications often compounding existing inequities while frustrating efforts to bring about change. For instance, local authority complaint systems were reportedly avoided on grounds of being both time and energy consuming.

Time can be unseen and intangible and thereby easily ‘invisibilised’ within the care system. Stuart, a gay man in his early forties who had moved in to care for his mother, kept a diary of his experiences for us. As his mother’s reliance on the input of formal care increased, so did the demands on Stuart’s time as he struggled to coordinate the different aspects of care. For instance, he described a protracted episode, acting as an intermediary between public and third sectors over delayed payment for his mother’s support. After weeks of telephone calls to both agencies he notes the imperious tone that meets his efforts to explain the predicament:

The [charity] admin person remains unmoved. [Their] head office are hassling her over the non‐payment and she, in turn, is heaping the hassle onto my shoulders… I point out the stress this on‐going situation has caused me – stress compounded by the on‐going health issues of my mother… and I am offered a detailed insight into what the [charity] admin person would have done were it her mother in this situation.

In this way care partners were coerced into a brokerage role in a system of fragmenting services and sectors. Such time‐consuming responsibilities were additional to the demands of caring and had the effect of colouring a person’s affective experience of time passing, eroding already limited discretionary time.

We have identified here just some of the ways access to formal care and support ‘takes time’ from people living with dementia and unpaid carers but the key insight concerns the cumulative nature of these temporal demands. Understood collectively and cumulatively the overall impact is to erode an entire networked system of reciprocated care, support and investment of time that encircles caring relationships. Not only have the risks of under‐occupation and isolation for people living with dementia remained but the time pressures on carers have intensified rather than been ameliorated through service delivery.

## IMPLICATIONS AND RECOMMENDATIONS

Practitioners are often a focus for recommendations for change to health and social care. Yet through accounts from people living with dementia and carers, we discovered that workers face their own struggle with time as a condition of their employment and are granted minimal latitude for change. Their own time pressures force ethical tensions to materialise in the form of missed visits, late arrivals/early departures and the pace at which they are required to work. In some instances, practitioners are temporal mediators, acting as a buffer on behalf of those they support, even becoming accomplices in an effort to subvert the dominance of service time and the meagre temporal affordances it allows. Such insights suggest a need to target change upstream through a focus upon what Sharma (2014) calls the ‘temporal architecture’ of the care system, that is, the mix of technologies, commodities, policies and practices that function to uphold ‘structures of power that drain, tire and exploit people’s time’ (p. 139). Our findings suggest three areas and related outcomes of the temporal organisation of care that warrant closer scrutiny.

We have shown the potential for the lived experience of time to serve as a barometer of the quality of care and support. Our findings reveal the significance of the embodied and visceral experience of time passing, of the way time sometimes refuses to flow but instead pools up for those left to wait or with little to do and of the sense of ‘non‐stop inertia’ (Baraitser, 2017) for carers who continually struggle to keep up. We saw how the care and welfare system can create a frustrated sense of urgency instilled in people awaiting payments to be made and how people were dogged by trepidation, enduring a slow anxious wait while stuck in the in‐between time of pending decisions or assessments. These ways of being in time are shared by people accessing formal care, but rarely acknowledged or made visible, let alone acted upon. We argue that such experience should be a consideration for service providers, how time feels could and should inform care, figuring in debate over wellbeing and quality of life.

A focus on time told a particular story about the absence of influence or control over care, for people living with dementia and their care partners. The beginnings and endings of support, it’s duration, frequency and day‐to‐day fluctuations due to backlogs and delays all point to the dominance of service time. Such conditions position people as ‘end users’ of a pre‐determined service and directly contradict recent policy ambitions for greater self‐directed support, co‐production in the design and delivery of services, person‐centred practice and ‘user‐defined’ outcomes. We need to look at ways that funding models and care practice can promote greater control over time collectively for people with dementia as well as at the individual level. Indeed, the flexibility to allow a degree of temporal autonomy should be central to translating person‐centred theory into practice.

We also saw how the care system contributed to a broader process of the social ordering of temporal worth. Service delivery repeatedly sends messages to people with dementia and unpaid carers as to the value of their time. Unavailable practitioners, prolonged waits for decisions on support and continual rationing carry messages about the worth of the person, not just their time, and highlights ethical questions associated with care provision. Our findings suggest the care system can reinforce and amplify existing inequalities, compounding the precarity that many face often as a feature of living with chronic illness or caring. We need to make visible the health, wellbeing, social and subjective impact of the under‐timing of care. This includes considering ‘time ethics’ when workers import their own time pressures into the home of a person with dementia. Additionally, there is a role for providers to map the temporal entanglements between formal and informal support in people’s lives, using this to adopt an integrating and bridging role instead of leaving service users themselves to act as brokers and intermediaries.

## CONCLUDING REMARKS

Beyond biomedical narratives, time has been overlooked and under‐theorised in dementia studies. So, what does it mean to think temporally about dementia? We argue for understanding time as fundamentally political and relational with social and material consequences. This includes attending to the lived experience of time: its embodiment and affectivity; the quality of time‐use and the multiplicity of temporalities. We have shown that social exclusion operates temporally; indeed, a focus on temporality helps to capture the unfolding and shifting experience of disadvantage (and privilege) in the lives of people with dementia. The experience of time is identity‐forming, both integral to our social situation and produced by it. As Baraitser (2017) shows, even the language of time offers helpful alternatives to more commonplace spatial metaphors which conjure a static field of power through cartographic approaches to inequality. Consequently, a focus on time (and talk of time) has the potential to animate our understanding of in/dependence and dis/ability in ways that disrupt existing conceptions of dementia.

We have demonstrated that time binds people, and how one person’s time use and losses inevitably and unavoidably shape the temporalities of others. Caught in a cycle of struggling to make time, while continually having time taken, unpaid carers share an impoverishment of discretionary time. Informal support networks can be vital redistributive mechanisms but time‐taking, in the form of rationing and reductions to formal support, ripple outwards across these networks. With a narrow focus on the individual, providers appear unaccountable for the secondary and tertiary outcomes to their actions. Being caught within a fragmented care system requires bridging the gaps, which further drains time, despite policy emphasis on integration to create seamless support. Hence, accessing formal support is frequently disempowering, as illustrated by the erosion of (temporal) autonomy, with little scope to challenge or change these conditions. The feeling and experience of time, and quality of time use alongside questions concerning ‘enough time’, how this is judged and by whom, should be integral to how we define what makes care good.

People with dementia and unpaid carers have been disproportionately affected by a policy of austerity. Rationing and withdrawals of personal support are undermining local support systems, and are layered over broader impacts to transport, upkeep of neighbourhood spaces and accessibility of public services (see McGarry, 2018). We have shown that time, in all its different guises, can both illuminate and generate such inequity. Fundamental to questions of social justice and ethics, time is a site of ongoing material struggle for people affected by dementia. We have revealed how budgetary and resource cuts ‘take time’ from those least‐well disposed to lose it and from people whose time is precious in the context of a life‐limiting condition. A temporal lens reveals that an economic policy of fiscal consolidation has diminished the capacity for social care to mediate in questions of social justice and equality. Through a narrow focus on the individual, it has lost its wider social accountability at times compounding inequity. Perhaps by ensuring accountability to the collective (i.e. to the chosen neighbourhoods of people with dementia) as well as to the person, this can help reinstate the values, principles and diverse temporalities that belong at the heart of the care system.

## AUTHOR CONTRIBUTIONS

**Richard‐Ward**: Writing—original draft; Conceptualization; Data curation; Formal analysis; Funding Acquisition; Investigation; Methodology; Project Administration; Resources; Supervision; Visualization. **Kirstein Rummery**: Supervision (Equal); Writing—review and editing (Equal). **Elzana Odzakovic**: Data curation (Equal); Formal analysis (Equal); Investigation (Equal). **Kainde Manji**: Data curation (Equal); Formal analysis (Equal); Investigation (Equal). **Agneta Kullberg**: Data curation (Equal); Formal analysis (Equal); Investigation (Equal); Supervision (Equal). **John Keady**: Funding acquisition (Equal); Supervision (Equal). **Andrew Clark**: Conceptualization (Equal); Data curation (Equal); Project administration (Equal); Supervision (Equal); Writing—review and editing (Equal). **Sarah Campbell**: Formal analysis; Data curation; Investigation.

## CONFLICT OF INTEREST

The authors declare no potential conflicts of interest with respect to the research, authorship and/or publication of this article.

## ETHICS STATEMENT

Ethical approval was obtained from the NHS Health and Social Care Research Ethics Committee (record reference 15/IEC08/0007) and the Regional Ethical Review Board in Linköping (the county of Östergötland, Sweden) (record reference 2013/200‐31 and 2014/359‐32) as well as relevant institutional approval.

## CONSENT

All participants gave written or recorded consent to participate and had capacity to provide informed consent.

## Data Availability

A metadata record of the research programme is currently being prepared for submission to the UK Data Service.
